# Comparison of the Meibomian Gland Openings by Optical Coherence Tomography in Obstructive Meibomian Gland Dysfunction and Normal Patients

**DOI:** 10.3390/jcm9103181

**Published:** 2020-09-30

**Authors:** Xinhan Cui, Qingfan Wu, Zimeng Zhai, Yujing Yang, Anji Wei, Jianjiang Xu, Jiaxu Hong

**Affiliations:** 1 Department of Ophthalmology and Visual Science, Eye, and ENT Hospital, Shanghai Medical College, Fudan University, 83 Fenyang Road, Shanghai 200031, China; xinhan.cui02@fdeent.org (X.C.); qingfan.wu@fdeent.org (Q.W.); zimeng.zhai@fdeent.org (Z.Z.); yujing.yang@fdeent.org (Y.Y.); anji.wei@fdeent.org (A.W.); jianjiang.xu@fdeent.org (J.X.); 2 Lanhe Optometry, 600 Middle Longhua Road, Shanghai 200032, China; 3 Department of Ophthalmology, The Affiliated Hospital of Guizhou Medical University, Guiyang 550025, China; 4 Shanghai Key Laboratory of Visual Impairment and Restoration, Science and Technology Commission of Shanghai Municipality, Shanghai 200031, China; 5 Key Laboratory of Myopia, Ministry of Health, Shanghai 200031, China

**Keywords:** meibomian gland dysfunction (MGD), meibomian gland terminal duct, meibomian gland orifice, anterior segment optical coherence tomography (AS-OCT), morphology, pathology

## Abstract

Purpose: The aim of this study was to use swept-source anterior segment optical coherence tomography (OCT) to explore imaging the meibomian gland openings and to identify their in vivo characteristics in patients with obstructive meibomian gland dysfunction (MGD) and healthy participants. Methods: We enrolled 49 patients with MGD and 54 health controls in this case-control study. Each participant underwent slit-lamp examination, meibography, and OCT scanning. Sixteen patients with MGD underwent a repeat OCT examination after eyelid massage. The outcome measures included determinations of meibomian gland openings (orifices and terminal ducts) from OCT images and comparisons of the meibomian openings between patients with MGD and normal controls before and after meibomian gland massage. Results: Using the same OCT scanning model, the number of visible orifices of the meibomian glands was similar between eyes with MGD and normal eyes (9.2 ± 2.3 vs. 9.7 ± 2.4). The mean diameter of the terminal ducts in patients with MGD was larger (120.22 ± 27.92 µm vs. 100.96 ± 20.30 µm) than in the normal controls, and had a larger coefficient of variation. Significant differences were observed in the mean diameter of the terminal ducts of patients with MGD before and after meibum gland massage (133.73 ± 27.81 μm vs. 102.26 ± 24.30 μm, *p* < 0.001). Conclusions: Patients with MGD have more diversified orifices and larger meibomian gland terminal duct diameters than normal subjects. In addition, meibomian gland terminal duct diameters seem to decrease in patients with MGD after meibum gland massage.

## 1. Introduction 

Meibomian gland dysfunction (MGD) is characterized by functional abnormalities of the meibomian glands [1]. MGD is likely the primary cause of evaporative dry eye and may occur in conjunction with aqueous-deficient dry eye [2,3]. Anatomically, MGD is characterized by gland orifice obstruction, duct dilation, atrophic degeneration of the gland, and Meibomian gland loss [3]. Meibomian gland orifice closure is thought to attenuate the delivery of meibum to the tear film, leading to excessive evaporation of the tear film and subsequent ocular surface disorders [4,5]. Hence, investigating the histological structure of the meibomian gland opening is important for the diagnosis and treatment of MGD. 

Previously, slit-lamp measurements were the only method for evaluating the meibomian gland orifices in vivo. Pictures might be helpful for depicting the hyperemia of the eyelid, clogging of the meibomian orifices, and anomalies of conjunctiva or eyelash, but it cannot be used to measure the gland opening diameter [6]. Noncontact infrared meibography is an effective method used to detect the morphology of meibomian glands, revealing abnormalities like dropout, distortion, shortening, and thickening [7]. However, the method focuses on the general shape of meibomian glands, with limited effect on displaying the patterns of orifices. The introduction of in vivo laser scanning confocal microscopy (IVCM) has addressed these issues by providing information on acinar density and diameter and gland orifice features [8,9]. However, IVCM is limited by its cross-sectional scanning mode and working distance, so the diameter of the terminal ducts of the meibomian gland is difficult to measure using IVCM. In addition, researchers labeled meibomian glands in both human eyelid and human cadaver eyelid, revealing that the acinar pictures captured with IVCM in most of studies were rete ridges in the dermal-epidermal junction, not meibomian glands [10]. Recently, optical coherence tomography (OCT) was introduced for the in vivo assessment of the morphology of the meibomian glands to provide depth information and enhanced visibility of the acini and duct, providing a fast and convenient method to quantitatively and volumetrically evaluate the meibomian glands [11,12]. To the best of our knowledge, no study has yet used OCT to determine meibomian gland opening, including the orifices and terminal ducts. The aim of the present study was to elucidate the anatomic details of meibomian gland openings based on OCT imaging, and to explore the changes in these openings after meibum gland massage.

## 2. Materials and Methods

### 2.1. Participants

Participants included in this study were enrolled between January and March of 2018 in a single tertiary eye center, Shanghai Eye, Ear, Nose and Throat Hospital of Fudan University (Shanghai, China). Written informed consent was obtained from all subjects before examination, and this study adhered to the tenets of the Declaration of Helsinki. The institutional review board of the hospital approved this project (IRBEENT-2018-03-103).

Patients with obstructive MGD were defined as those with altered tear film, irritated eye symptoms, terminal meibomian duct obstruction, and/or qualitative/quantitative changes in the glandular secretion [3]. Participants were excluded if they had any ocular disease other than dry eye disease or any ophthalmic surgery history. If both eyes met the eligibility criteria, one eye was selected randomly. Overall, 49 eyes from 49 patients with MGD were enrolled in the MGD group. After the collection of demographic information and ophthalmic history, each participant underwent best-corrected visual acuity (BCVA) measurement, slit-lamp examination, and completed an ocular surface disease index (OSDI) questionnaire. Subjects were enrolled only if they had normal BCVA (better than 20/25) and an absence of other ocular diseases. They then underwent sequential lid margin photography, meibography, and anterior segment OCT. The controls included 54 eyes from 54 age- and sex-matched volunteers. The participants in the control group had no complaint of ocular surface irritation, and without any anterior segment abnormality from slit-lamp examination. Tear break up time, fluorescein staining, and Schirmer 1 test without anesthesia were taken as the screening test to ensure no dry eye patient would be recruited into the control group.

Of the 49 patients with MGD, 26 eyes of 16 patients had obviously clotted meibomian glands or enlarged meibomian ducts, and were accepted to receive meibomian gland massage, followed by a repeat of the OCT examination 30 min after the treatment.

### 2.2. Meibography

In this study, Oculus Keratograph (Oculus GmbH, Wetzlar, Germany) was selected for the meibography in the study subjects. This machine uses infrared (IR) light, and is considered a noninvasive imaging device with automated features that do not require topical anesthetic, fluorescein staining, white light, or manual timing [13]. Following upper eyelid eversion, the Oculus K5M meibography tool was used to generate IR images of the tarsal conjunctiva. 

### 2.3. Swept-Source Anterior Segment Optical Coherence Tomography

A Casia SS-1000 OCT instrument (Tomey, Nagoya, Japan) with a swept-source laser wavelength of 1310 nm was chosen to image the meibomian gland openings in this study. The bleb segment mode was selected to picture the filter bleb after glaucoma surgery, which performs a horizontal or vertical raster scan in 8 × 8 mm areas, with a depth of 6 mm. In this study, this mode was chosen to evaluate the morphology of the meibomian gland opening. Each participant was asked to continue looking down during the scan. With the technician’s help, the upper lid was elevated gently to make the upper lid margin perpendicular to ground and to position the meibomian gland openings as horizontal as possible. Each eye was imaged three times. For repeatability, the middle of the upper lid of each subject was set as the middle point in this procedure. 

**Figure 1:**
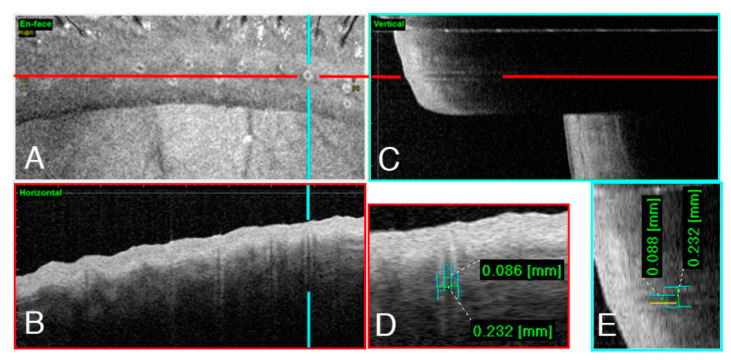
(**A**) En-face image of an eyelid margin scanned by Anterior Segment Optical Coherence Tomography (AS-OCT). Red and blue lines indicate the position of (**B**) horizontal and (**C**) vertical lining scans with maximal tube diameters, respectively. (**D**,**E**) The inner and outer diameters of the meibomian openings from horizontal and vertical images.

After the scan, an en-face image of the eyelid was automatically generated, and this clearly showed the number and morphology of the meibomian gland orifices in the eyelid margin (Figure 1A). Every operation included 256 horizontal (Figure 1B) and 256 vertical (Figure 1C) line scans in the chosen area. Determining the maximum diameter of the meibomian gland terminal ducts, either horizontally or vertically, was easy at this point. The OCT image of a meibomian gland opening resembles a Pasteur pipette, with the terminal duct serving as its main body and tapering to a narrow point (orifice) that opens onto the lid margin. The tube diameters of the meibomian terminal glands were determined by manually measuring the maximum diameters of each duct in both horizontal and vertical directions, and then averaging the values (Figure 1D,E). In the same eye, every terminal duct of the gland in the chosen area was measured and averaged, which means that every eye had 6–12 values for the maximal duct diameters. These values were then analyzed statistically, and the average, maximal, and minimal diameters and the variation of the diameters were calculated. 

Briefly, the en-face image provided one measurement: the number of meibomian orifices in the upper eyelid with a length of 8 mm. The OCT image that was aligned with the maximum duct diameter on infrared imaging offered 2 measurements: the maximal inner and outer diameter of the meibomian terminal duct diameters. When combined with the en-face image and line scanning pictures, every meibomian gland opening detected by OCT was measured for its inner duct diameter, both horizontally and vertically. Based on descriptive statistics, 4 characteristics were derived: (1) average diameter (Mean), (2) the maximal diameter (Max), (3) the minimal diameter (Min), and (4) the variation of the diameter (standard deviation, Var).

A better understanding of the morphology of the meibum openings was obtained by collecting two pieces of eyelid margin from an eye bank, sectioning these vertically, and staining with hematoxylin and eosin (H&E).

### 2.4. Meibomian Gland Compress

The basic treatment for obstructive MGD is warm compresses and lid hygiene. We recommended that every patient use a meibomian gland compress in our clinic. However, some of them were afraid of pain; some chose lid hygiene alone as the treatment. The patients with severe clotted meibomian glands were strongly advised to apply the lid compress. In total, 16 subjects in the MGD group underwent meibomian gland compresses after the examination. After application of topical anesthesia, the patients were asked to lie down on the treatment table. The eyelid was everted and the meibomian glands of the upper eyelid were then gently squeezed with forceps. Topical antibiotics were applied after the treatment. All the treatments were performed by one practitioner. After a 30-min rest, the patients were asked to undergo another OCT examination. 

### 2.5. Statistical Analysis

Demographic and other baseline characteristics were summarized as means and standard deviations for continuous variables, or as frequencies for discrete variables. The differences between the MGD group and healthy controls were calculated with the Student’s *t*-test. A paired *t*-test was performed to investigate the differences in meibomian opening diameters before and after application of the massage. Pearson’s correlation test was used to determine the relationship between the meibomian tube diameters and other factors.

## 3. Results

The patients with MGD had a median age of 51 years (range: 24–72 years), including 36 (73.5%) women and 13 (26.5%) men. The median age of healthy volunteers was 46 years (range: 23–82 years), including 34 (63%) women and 20 (37%) men. 

### 3.1. Morphology of the Meibomian Gland Openings 

**Figure 2:**
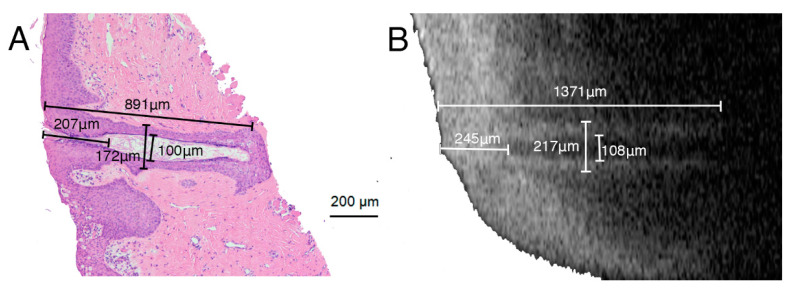
Comparison of (**A**) a photomicrograph of hematoxylin and eosin (H&E)-stained eyelid margin tissues and (**B**) anterior segment optical coherence tomography (OCT) image and showing the meibomian gland opening structures.

In this study, we measured the openings of meibomian glands in vivo with OCT, and compared these measurements with H&E-stained lid margin slides. The meibomian terminal duct tapered to a narrow point, and then opened onto the lid margin (Figure 2). Four indexes were measured for description: (1) the length of orifice, (2) the length of the whole terminal duct, (3) the maximal inner diameter of terminal duct, and (4) the maximal outer diameter of terminal duct, which also included the thickness of the glandular epithelium. The inner maximal duct diameter could be easily detected, since the OCT images were presented as three-dimensional data. As shown in Figure 2, these four characteristics are comparable in the histopathological sections and the OCT images. The relatively small values of these four indexes in the histopathological slides when compared with the OCT images may have occurred due to the dehydration during the H&E staining. In addition, the length of the orifice and the whole terminal duct may vary because the scan direction may be different. 

### 3.2. Comparison of Meibomian Gland Openings in Patients with MGD and Normal Subjects

**Figure 3:**
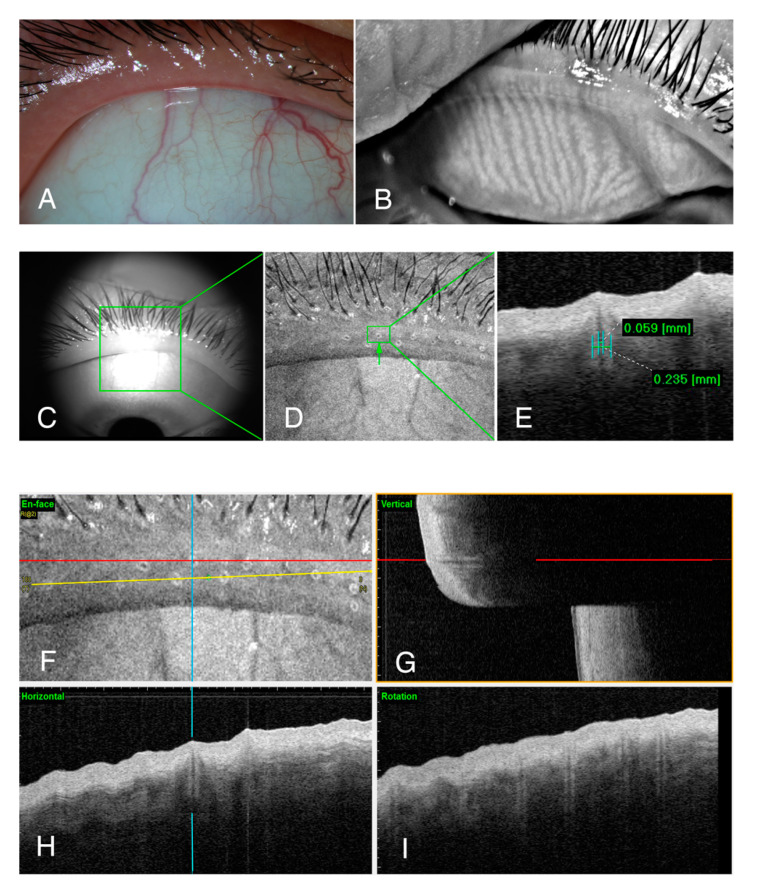
The eyelid margin and meibomian gland openings in normal eyes. Each participant was recorded by obtaining a photo of the upper eyelid margin (**A**), conducting meibography of the upper eyelid (**B**), and then scanning by OCT (**C**–**I**). During the OCT examination, an infrared picture of the chosen eyelid margin (**C**) was captured. The chosen area (green box in (**C**)) could then be transformed into an en-face image (**D**) that clearly showed every orifice (green box in (**D**)) in the eyelids. The inner and outer maximal diameter of every chosen meibomian terminal duct connected to the orifice (green box in (**D**)) can be measured (**E**). In the en-face image (**F**), three lines can be dragged to show the vertical (blue line (**G**)), horizontal (red line (**H**)), and rotational (yellow line (**I**)) sections.

**Figure 4:**
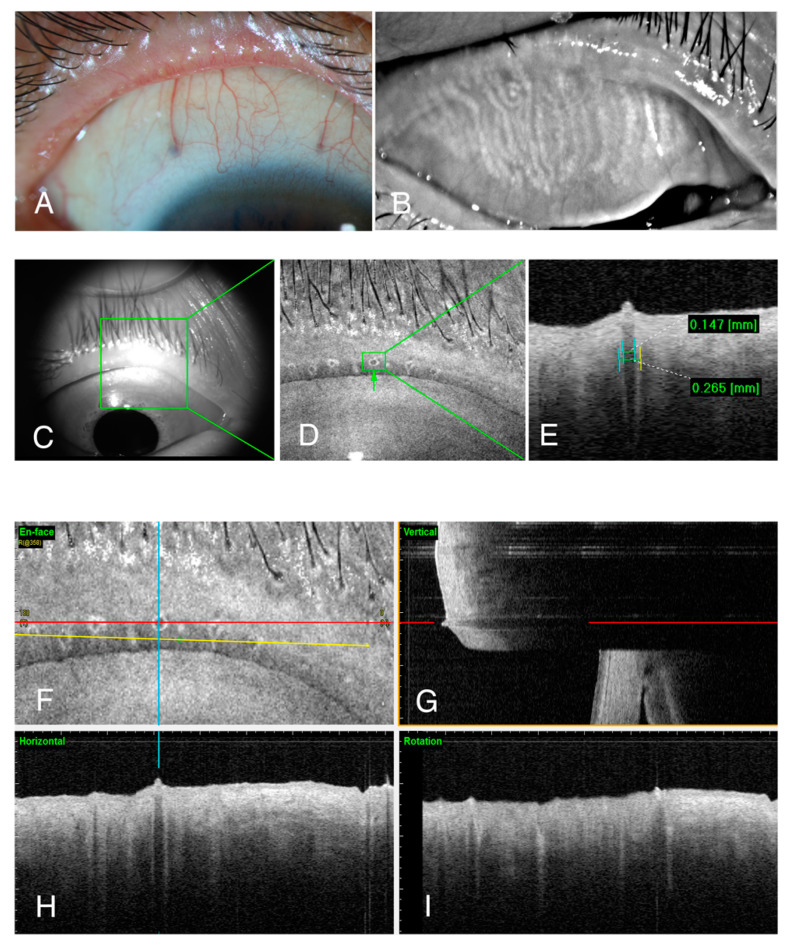
The eyelid margin and meibomian gland openings in patients with meibomian gland dysfunction (MGD). Each participant in this study underwent an eyelid margin photo (**A**), meibography of the upper eyelid (**B**), and an OCT examination (**C**–**I**). The chosen area for examination (green box in (**C**)) shows enlarged orifices (green box in (**D**)) and ducts (**E**). The diameter of both inner and outer maximal diameter of the terminal duct (**E**) can be numbered both vertically (**G**) or horizontally (**H**). With the help of the rotation line (yellow line in (**F**)), any two of the tubes can be compared in the same picture (**I**).

Healthy eyes with clear orifices (Figure 3A) and uniform meibomian glands (Figure 3B) had meibomian gland openings with relatively small but uniform orifices (Figure 3D) and terminal ducts (Figure 3F–I). Contrastingly, the OCT images of patients with MGD with opaque meibum (Figure 4A) and irregular meibomian glands (Figure 4B) indicated the substantial enlargement of some of the orifices (green box in Figure 4D), and an apparent blockage of the head of the opening (Figure 4E). The terminal meibomian ducts were also enlarged, with a diameter often two to three times that of normal ducts (Figure 4E,G,H). Although perhaps not as obvious in the orifices, the diameters of the meibomian terminal ducts varied widely in the patients with MGD. With skillful rotation (yellow line in Figure 4F), any two of the duct diameters could be observed simultaneously and compared directly (Figure 4I). 

The numbers of orifices and the maximal diameters of the meibomian gland terminal ducts were measured, processed, and compared between the two groups (Table 1). The numbers of orifices did not differ statistically between patients with MGD and their healthy counterparts. However, patients with MGD mostly had larger (Mean and Max in Table 1) and more varied (Var in Table 1) diameters of the meibomian gland terminal ducts.

### 3.3. Changes in Meibomian Gland Openings in Patients with MGD before and after Massage

**Figure 5:**
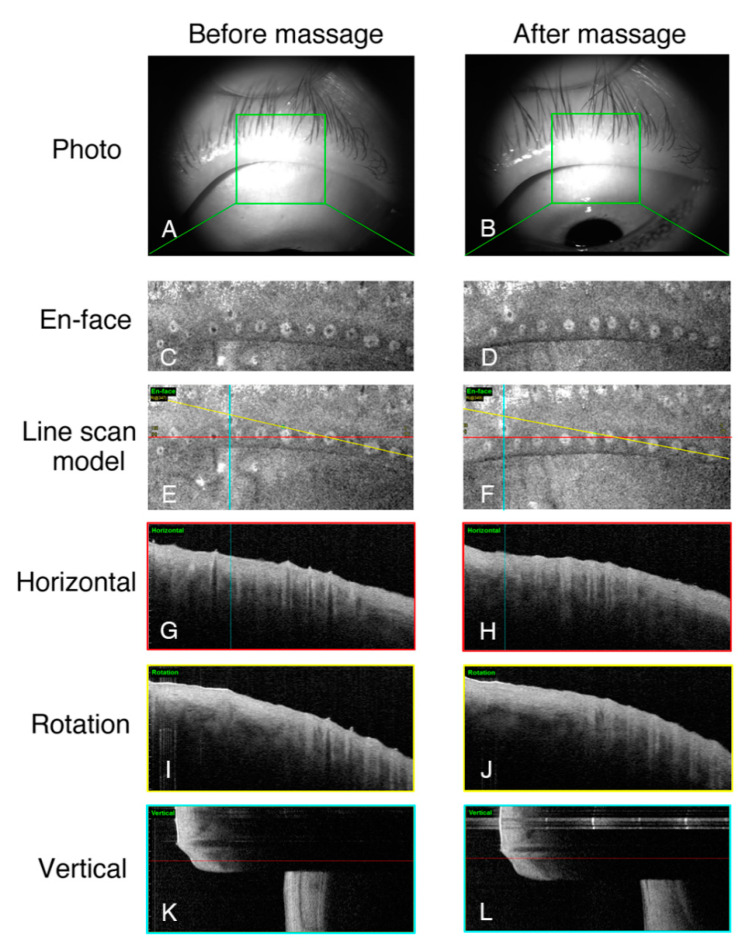
The changes in the meibomian gland openings before (**left**) and 30 min after (**right**) meibomian massage. In this study, we chose the same area for examination to the best of our abilities (**A**,**B**). The changes in the characteristics of the orifices are clearly shown in the en-face images (**C**,**D**). With the help of a line scan (**E**,**F**), the same terminal gland ducts in two examinations can be compared horizontally (**G**,**H**), rotationally (**I**,**J**), or vertically (**K**,**L**).

The 26 eyes of 16 patients MGD patients underwent meibomian gland massage after enrollment, and then underwent an additional OCT examination 30 min later (Figure S1). Figure 5 shows a representative case where following squeezing out of the meibum, the shrinking of the orifices (Figure 5C,D) and terminal ducts (Figure 5G–L) was observed. Statistical analysis confirmed the change in the mean diameter of the terminal ducts of patients with MGD before and after meibum gland massage (133.73 ± 27.81 μm vs. 102.26 ± 24.30 μm, *p* < 0.001). In addition, the maximal terminal duct diameter was reduced by nearly 30% after massage from 271.02 ± 114.78 µm to 193.50 ± 70.24 µm (*p* < 0.001; Table 2), while the degree of variation decreased from 69.43 ± 39.81 µm to 45.20 ± 21.18 µm (*p* = 0.001; Table 2). 

## 4. Discussion 

Meibomian gland dysfunction is the leading cause of evaporative dry eye and is also the most common pathology underlying cases of aqueous-deficient dry eye [1,14]. The meibomian glands are recognized as large sebaceous glands vertically aligned in the eyelids, but research into their morphology related to their openings is limited. Our findings demonstrated the value of OCT for identifying the orifices and terminal ducts of the meibomian glands. When compared with a histopathologic study of the lid margin, the OCT images are convincing and dynamic. Building a 3D model of the lid margin with a length of 8 or 16 mm is possible. This study aimed to characterize the morphology of the meibomian gland openings and to offer a noninvasive method to measure the diameter of the meibomian gland terminal duct. In addition, we determined the effect of meibum gland massage on the orifices and meibomian terminal ducts, so the OCT images also offer a new method to evaluate the response to MGD treatments. 

Meibomian gland orifices are visible in a slit-lamp examination, which is usually performed to evaluate the meibum quality and meibomian gland activity [15]. With high resolution cameras, the details of eyelids can be easily captured and presented. The hyperemia of eyelid, clogging of orifices, and abnormality of eyelashes can be clearly detected with this examination. However, in MGD patients with cloudy expressed meibum or hyperemic marginal conjunctiva, these orifices become obscured and are difficult to identify. OCT allows the orifices to be numbered and characterized. The OCT pictures obtained can be easily measured and calculated, providing more value in the assessment of MGD prognosis and establishing discrimination criteria [12].

The upper eyelid contains about 25–40 glands [16]. We found that 9.74 ± 2.36 orifices exist in the middle 8 mm of the upper eyelid (Table 1), and this number was not statistically different in patients with MGD. Even in the dropout meibomian glands determined by meibography, the orifices and terminal ducts were still evident (Supplemental File 1), suggesting that OCT-based meibomian gland assessment might provide additional insights into MGD pathogenesis. The number of orifices in severe obstructed MGD patients slightly increased after meibomian gland massage. We suppose that in these patients, some of the orifices and terminal glands filling with cloudy meibum would be obscure and hard to distinguish, so meibomian gland compress made them easier to find (Supplemental File 2). However, it was impossible to consider all of the glands, so this result suggests that the orifices being measured differed. Further studies should be conducted to find the answer to this question. The duration of glands change produced by compress should also be examined. 

The meibomian gland terminal ducts, which are about 0.5 mm long, are the final excretory tubes, and are surrounded with cornified epithelium [4,17]. In this study, the examination depth of the OCT scan was often more than 1 mm, which guaranteed a thorough examination of the terminal ducts. The maximal duct diameter in healthy participants in this study was close to 150 µm, and this value was nearly 40% higher in patients with MGD (Table 1). The enlarged terminal ducts and orifices might be caused by meibum obstruction, which is the most common form of meibomian gland dysfunction [4,18]. Since the dysfunctional glands are enlarged by obstruction, the obstructed duct diameters are larger than those of unaffected ducts. In the current study, the terminal duct diameters in patients with MGD also showed wider diversity (Table 1). Conversely, the removal of the obstruction by meibum gland massage quickly reduced both the mean and the variation of the maximal duct diameter to the normal level (Table 2). 

An animal model of obstructive MGD showed that the early changes in meibomian glands after orifice closure cannot be detected by meibography [19], whereas clinical research indicated that meibum quality is the most commonly used variable for evaluating the changes after MGD treatment [20]. Taken together, our data suggest that OCT might be a novel and valuable alternative tool for the early diagnosis of MGD and for follow-up evaluations after MGD treatment.

This study had several limitations. First, the sample size was small, and our patients with MGD were not classified, either by pathogenesis or severity. Observation of the orifices and terminal duct morphology alterations in different types of MGD might be of interest in future studies. Second, we chose the middle part of upper lid margin as the target of OCT examination for purposes of repeatability. However, the terminal meibomian glands in the peripheral part of upper lid margin and in the lower lid margin were left unexplored by this choice. Still, the chosen middle part of openings brought the selection bias into consideration. In addition, topical anesthetic may have an effect on meibomian gland size, which could bias our investigation as well. To date, few reports has discussed this potential effect, but we think that it would be interesting to raise this question in future research.

## 5. Conclusions

Our findings showed that OCT imaging can be a valuable tool for investigating the morphological changes in meibomian gland openings in vivo. We showed that patients with MGD often have enlarged orifices and dilated terminal ducts compared to normal subjects, and these parameters are improved by meibum gland massage. A comprehensive evaluation of the meibomian gland openings could better clarify the role of the meibomian gland openings and eyelid margin anatomy on the pathogenesis of MGD, as well as facilitate the detection and follow-up of MGD.

## Figures and Tables

**Table 1:** The differences in meibomian openings between normal subjects and patients with meibomian gland dysfunction (MGD).

Variable	Control Group (*n* = 54)	MGD Group (*n* = 49)	*t*-Value	*p*-Value
Age (years)	46.35 ± 14.4	51.16 ± 12.97	−1.775	0.079
Sex	20 M/34 F	13 M/36 F	−1.142	0.256
Orifices ^1^	9.74 ± 2.36	9.24 ± 2.30	1.080	0.283
Mean ^2^ (μm)	100.96 ± 20.30	120.22 ± 27.92	−4.031	<0.001 *
Max ^3^ (μm)	157.89 ± 4.03	224.17 ± 94.47	−4.420	<0.001 *
Min ^4^ (μm)	59.82 ± 21.26	64.90 ± 18.94	−1.274	0.206
Var ^5^ (μm)	33.03 ± 15.81	51.70 ± 3.51	−4.252	<0.001 *

^1^ the numbers of meibomian gland orifices in the en-face images of optical coherence tomography (OCT) examinations; ^2^ the average of the maximal duct diameters (Mean); ^3^ the maximum value for the maximal duct diameters (Max); ^4^ the minimum value for the maximal duct diameters (Min); ^5^ the variation in the maximal duct diameters (standard deviation, Var); * *p* < 0.05.

**Table 2:** Changes in the meibomian gland openings in patients with meibomian gland dysfunction (MGD) before and after meibomian gland massage.

Variable	Before Massage	After Massage	*t*-Value	*p*-Value
Orifices ^1^	9.98 ± 2.74	10.54 ± 2.42	−2.527	0.018 *
Mean ^2^ (μm)	133.73 ± 27.81	102.26 ± 24.30	6.297	0.000 *
Max ^3^ (μm)	271.02 ± 114.78	193.50 ± 70.24	4.098	0.000 *
Min ^4^ (μm)	62.77 ± 22.55	53.81 ± 19.26	1.737	0.095
Var ^5^ (μm)	69.43 ± 38.91	45.20 ± 21.18	3.596	0.001 *

^1^ the numbers of meibomian gland orifices in the en-face image of optical coherence tomography (OCT) examination; ^2^ the average of the inner maximal duct diameters (Mean); ^3^ the maximum value of the inner maximal duct diameters (Max); ^4^ the minimum value of the inner maximal duct diameters (Min); ^5^ the variation in the inner maximal duct diameters (standard deviation, Var); * *p* < 0.05.

## Supplementary Materials

The following are available online at https://www.mdpi.com/2077-0383/9/10/3181/s1, Figure S1: The eyelid margin and meibomian gland openings in patients with MGD with dropout meibomian glands. The orifices are not very evident in slit lamp examination (A). In meibography, most of the meibomian glands were lost (B). When we used OCT to focus on the middle part of upper lid margin (C), all the orifices were still observed in the en-face image (D). The increased diameters of the terminal ducts can be measured in line scanning mode (E–I). Figure S2: The details of meibomian gland openings determined by OCT imaging before (left) and after (right) meibomian massage in 14 selected patients with MGD.

## Declarations

Ethics approval and consent to participate: The institutional review board of the Shanghai Eye, Ear, Nose and Throat Hospital approved this project. Written informed consent was obtained from all subjects; Consent for publication: Not applicable; Availability of data and materials: The datasets supporting the conclusions of this article are available from the corresponding author on reasonable request.

## Abbreviations

**OCT**	optical coherence tomography
**MGD**	meibomian gland dysfunction
**TBUT**	tear film break-up time
**IVCM**	in vivo laser scanning confocal microscopy
**BCVA**	best-corrected visual acuity
**OSDI**	ocular surface disease index
