# Supplementary material for: Comparison of the Meibomian Gland Openings by Optical Coherence Tomography in Obstructive Meibomian Gland Dysfunction and Normal Patients
Source: J Clin Med. 2020 Sep 30;9(10):3181. doi: 10.3390/jcm9103181 (PMC7601658; doi:10.3390/jcm9103181)
Supplement: Supplementary file 1 [file jcm-09-03181-s001.pdf]

Supplementary Materials

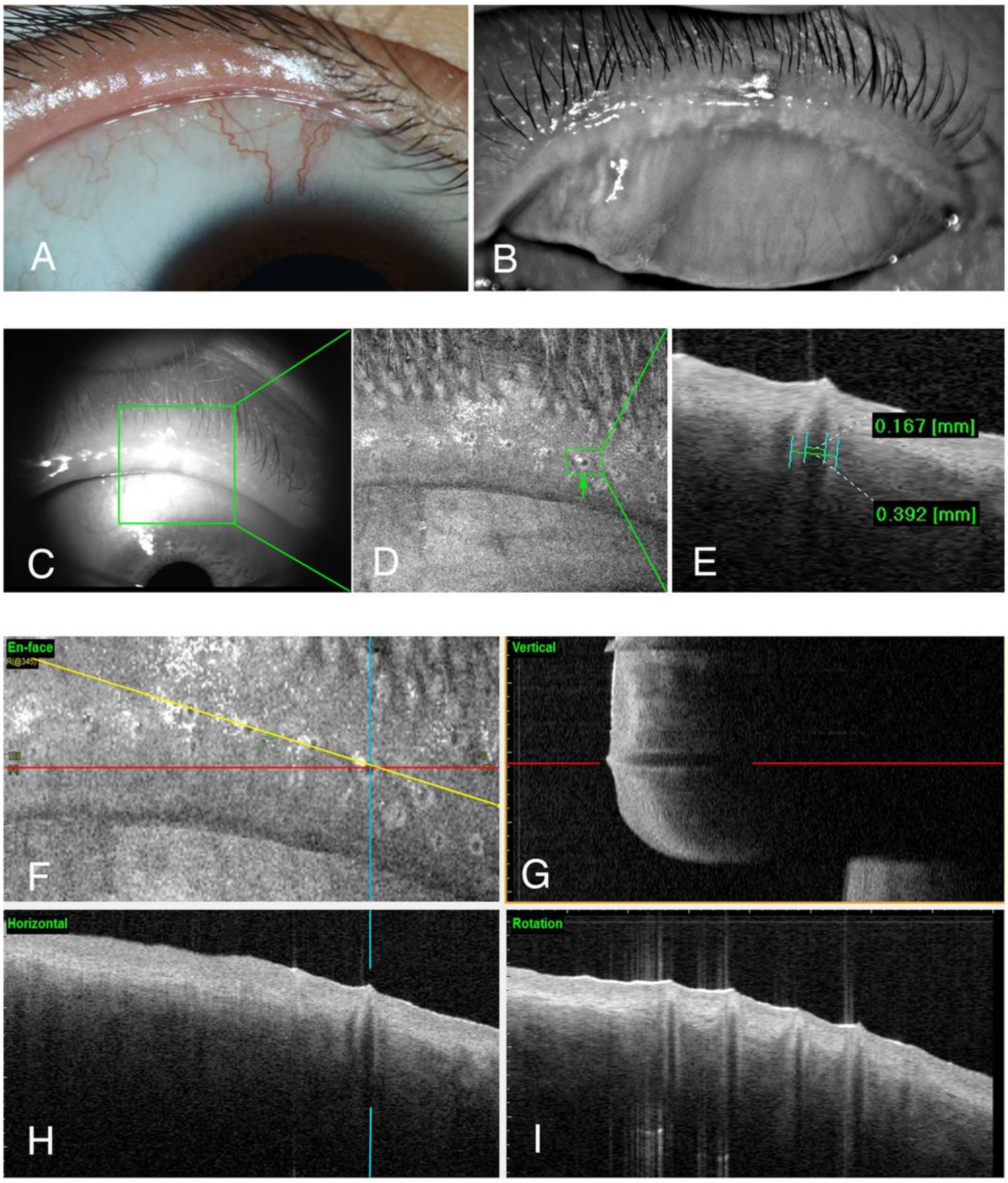

Figure S1. The eyelid margin and meibomian gland openings in patients with MGD with dropout meibomian glands. The orifices are not very evident in slit lamp examination (A). In meibography, most of the meibomian glands were lost (B). When we used OCT to focus on the middle part of upper lid margin (C), all the orifices were still observed in the en-face image (D). The increased diameters of the terminal ducts can be measured in line scanning mode (E–I).

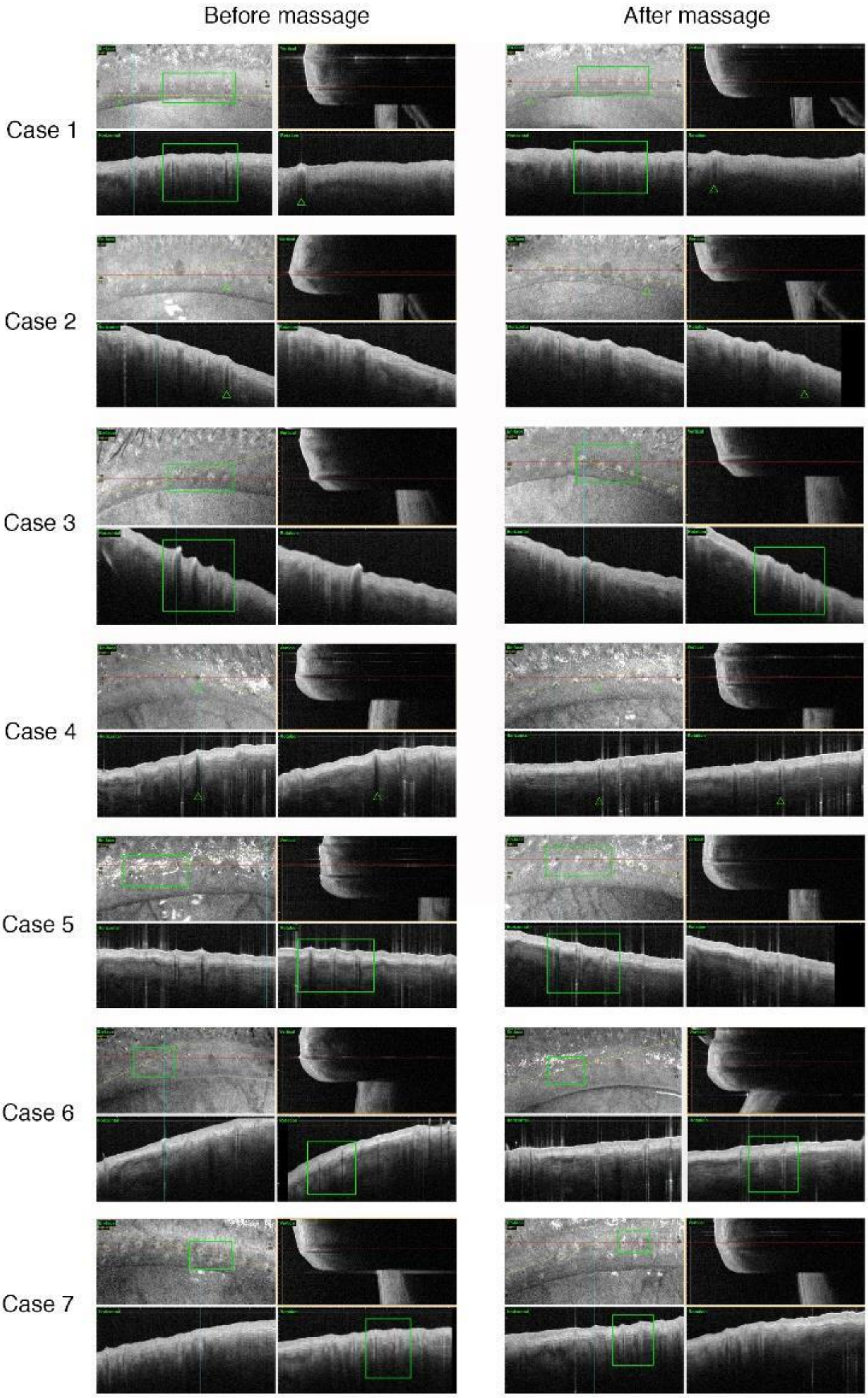

Figure S2. *Cont.*

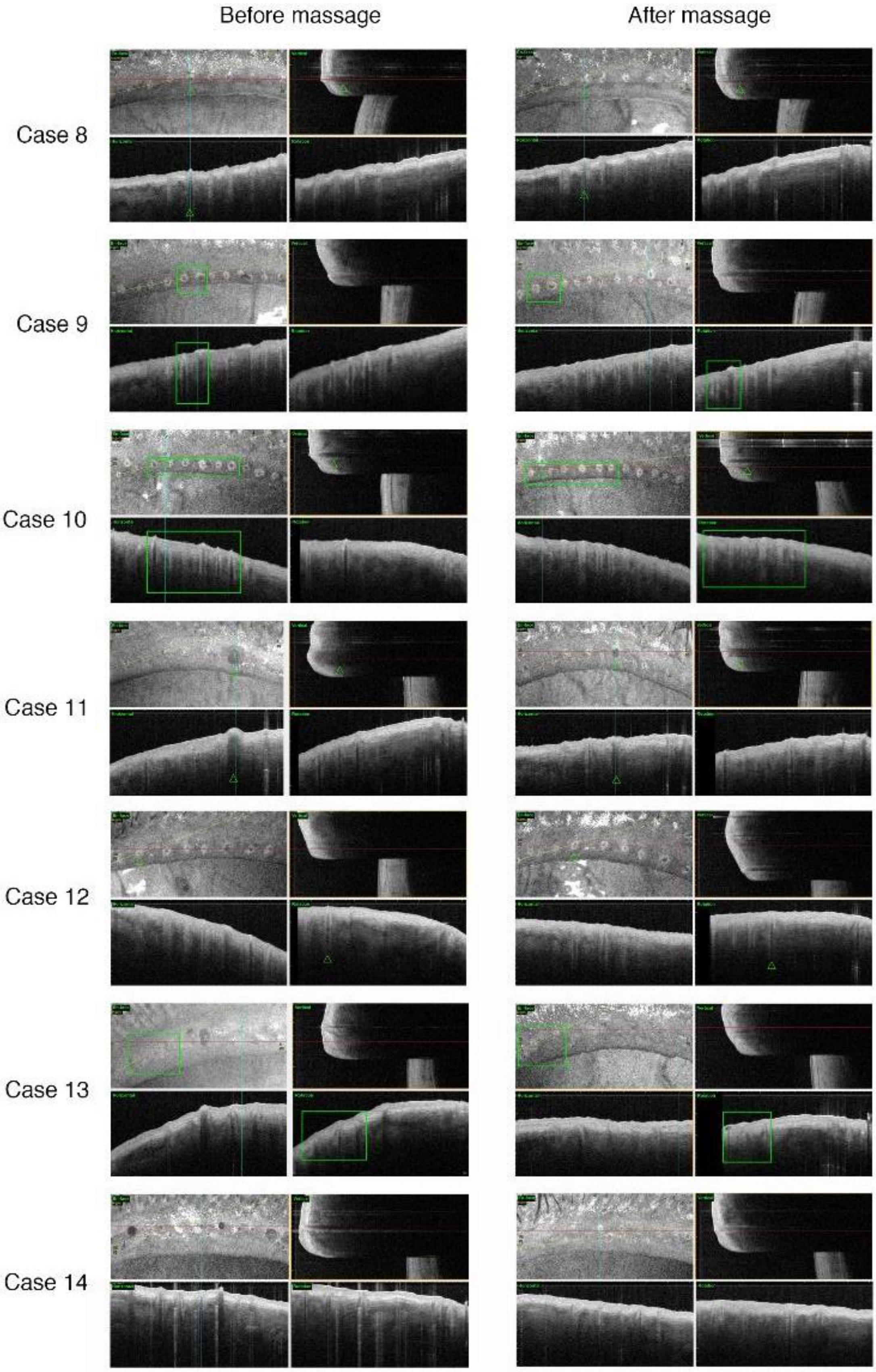

Figure S2. The details of meibomian gland openings determined by OCT imaging before (left) and after (right) meibomian massage in 14 selected patients with MGD.
